# DNA methylation variation along the cancer epigenome and the identification of novel epigenetic driver events

**DOI:** 10.1093/nar/gkab1167

**Published:** 2021-12-06

**Authors:** Richard Heery, Martin H Schaefer

**Affiliations:** Department of Experimental Oncology, IEO European Institute of Oncology IRCCS, Via Adamello 16, 20139, Milan, Italy; Department of Experimental Oncology, IEO European Institute of Oncology IRCCS, Via Adamello 16, 20139, Milan, Italy

## Abstract

While large-scale studies applying various statistical approaches have identified hundreds of mutated driver genes across various cancer types, the contribution of epigenetic changes to cancer remains more enigmatic. This is partly due to the fact that certain regions of the cancer genome, due to their genomic and epigenomic properties, are more prone to dysregulated DNA methylation than others. Thus, it has been difficult to distinguish which promoter methylation changes are really driving carcinogenesis from those that are mostly just a reflection of their genomic location. By developing a novel method that corrects for epigenetic covariates, we reveal a small, concise set of potential epigenetic driver events. Interestingly, those changes suggest different modes of epigenetic carcinogenesis: first, we observe recurrent inactivation of known cancer genes across tumour types suggesting a higher convergence on common tumour suppressor pathways than previously anticipated. Second, in prostate cancer, a cancer type with few recurrently mutated genes, we demonstrate how the epigenome primes tumours towards higher tolerance of other aberrations.

## INTRODUCTION

The epigenome is the complete set of epigenetic modifications within a cell, including DNA methylation and histone modifications, and plays a fundamental role in coordinating the emergence of tissue-specific patterns of gene expression and chromatin structure during tissue differentiation and development of multicellular organisms (1–3). Of these epigenetic marks, DNA methylation is the best characterized in mammals and serves a variety of functions, including genomic imprinting, X-chromosome inactivation in females, silencing of transposable elements and regulation of gene expression, with DNA methylation at gene promoters generally being associated with their transcriptional repression (4–6).

Tumours evolve under selection acting on alterations giving a growth advantage or disadvantage to the clones carrying those alterations. In tumour cells, the DNA methylation landscape is profoundly disturbed compared to their normal counterparts. Most tumours exhibit focal increases in methylation, particularly at CpG islands associated with gene promoters, against a background of genome-wide DNA methylation loss (7–9). Such changes in DNA methylation may occur during the very earliest stages of tumorigenesis, with alterations in DNA methylation observed even in pre-cancerous lesions associated with several tumour types (10–12). Yet, it is largely unclear which of these DNA methylation changes contribute to tumor initiation and progression and which are merely passenger events.

Hypermethylation of specific gene promoters and the associated repression of transcription has been recognized as a key mechanism of tumour suppressor gene (TSG) inactivation in cancer (13–15). In some cancers, genes may even be more frequently inactivated through promoter hypermethylation than by mutation (16,17). Notably, in sporadic tumours, somatic mutations in certain DNA repair genes, such as BRCA1 and MLH1, are quite rare; however, these genes are very often silenced through hypermethylation of their promoters (18). For example, hypermethylation of the MLH1 promoter appears to be responsible for most of the microsatellite instability (MSI) cases observed in sporadic colorectal, endometrial and gastric cancers, causally connecting perturbations in DNA methylation with the tumour mutation rate (19–21). Meanwhile, genes regulating DNA methylation, including DNMT1 and DNMT3A, the TET family and IDH1 and IDH2, are themselves frequently mutated in cancer and their mutation status is associated with changes to the cancer epigenome (22,23).

A number of different approaches have been developed to detect differential methylation between populations (24–27), though few of them are specifically designed for identification of differentially methylated regions in cancer. Accordingly, while a few studies have sought to catalogue the gene promoters which display altered DNA methylation in cancer (24,28,29), these efforts have struggled to distinguish which methylation events play an active role in cancer development from those which are most likely incidental and unimportant to tumorigenesis. This is analogous to the problem of separating driver and passenger mutations in cancer genomics.

A major confounder with studies seeking to discover mutated cancer driver genes has been the variation in mutation rates across different regions of cancer genomes, leading to the identification of many likely false-positive drivers (30). DNA methylation changes in cancer have also been shown to vary across the genome in a semi-predictable manner, with methylation loss often occurring in late replicating regions and methylation gain in regions of open chromatin (31–33). Thus, it is likely that a similar problem to that involving identification of mutated driver genes has undermined differentially methylated gene studies in cancer and led to the identification of many false positive epigenetic drivers whose altered methylation is likely merely due to their genomic location and incidental to tumorigenesis. Indeed, it has been noted that there are typically 1000s of differentially methylated genes in tumours, with only a tiny fraction of them overlapping TSGs and oncogenes (32). This raises the question which of those genes ultimately contribute to tumour initiation and progression by changes of their DNA methylation state.

**Figure 1.**
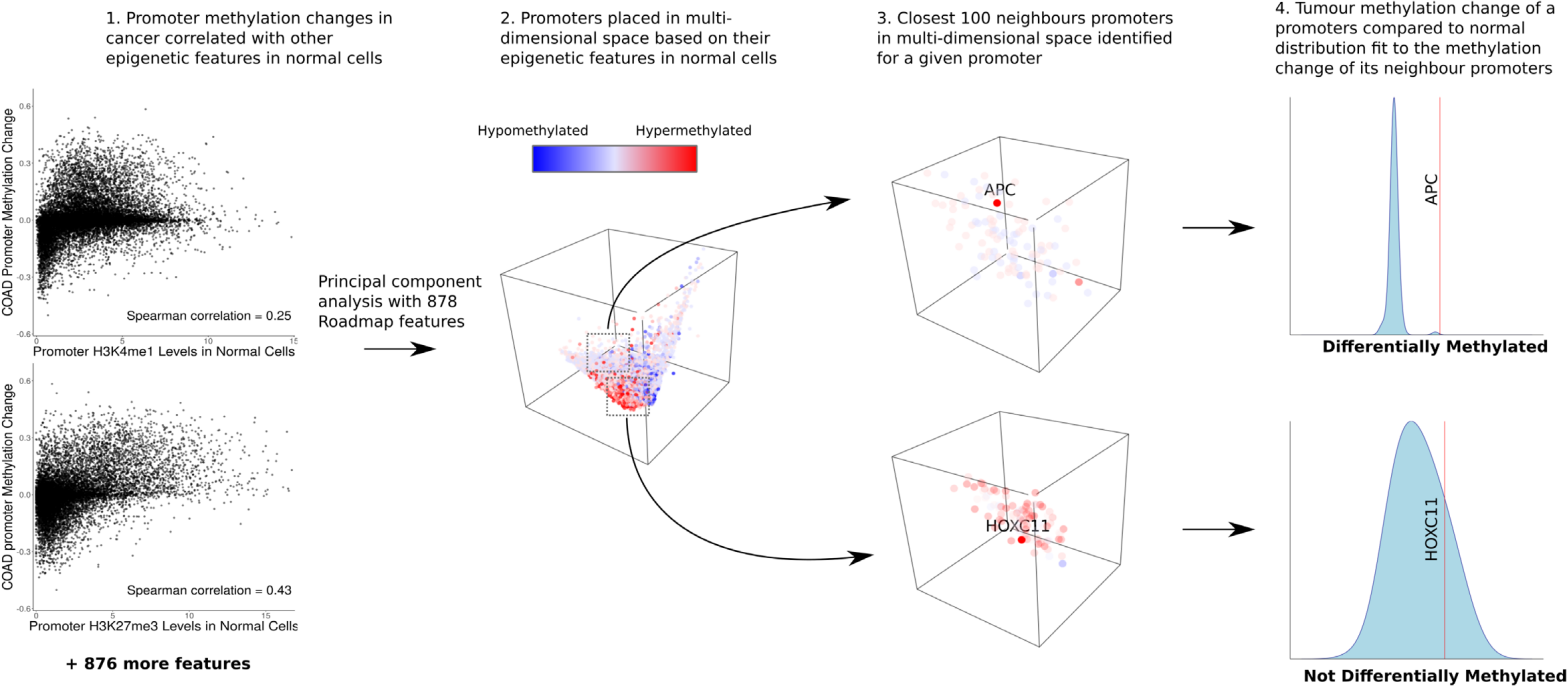
MethylDriver algorithm. Left-hand panels show association of promoter methylation change in colon tumour samples with their epigenetic features in healthy cells. Middle plot depicts the placement of promoters in a multivariate space using principal components (PCs) derived from the epigenetic promoter features in healthy cells and the subsequent identification of the 100 most similar neighbour promoters using Euclidean distance for two different promoters. The right-hand panels depict the fitting of normal distributions using the methylation change of a promoter's neighbourhood, leading to different outcomes for two gene promoters with a similar methylation change in colon adenocarcinoma: the TSG *APC* is affected by methylation change significantly greater than expected given its neighbourhood, while the change for *HOXC11*, a gene unexpressed in normal colon adenocarcinoma samples and that is significantly hypermethylated without correcting for epigenetic covariates, fits within what would be expected for its neighbourhood.

Thus, we present a computational tool, MethylDriver, to distinguish genomic regions whose differential methylation may play a causative role in cancer and can be considered putative epigenetic driver events from those which are likely not causally involved and can be considered epigenetic passenger events. MethylDriver accomplishes this by comparing the DNA methylation change in genomic regions between tumour samples and matched normal samples while taking account of the variation in the propensity for methylation change in different regions across the cancer genome. Like this we can directly quantify the excess of DNA methylation change compared to the background (i.e. expected) change of DNA methylation at a given promoter and thereby identify and eliminate spurious cases of expected DNA methylation loss or gain (Figure 1). We applied MethylDriver to 678 pairs of matched tumour and normal samples across 13 different cancer types from The Cancer Genome Atlas (TCGA) in order to identify putative driver methylation events at promoters.

## MATERIALS AND METHODS

### Promoter definition

Promoter genomic coordinates were downloaded for hg19 from the EPDnew promoter database (34). These promoters were resized to 1000 bp by adding the necessary number of bases upstream to the downloaded transcription start sites (TSS). We chose to use 1 kb as the promoter size as it is intermediate between promoter sizes used previously by other approaches to determine DNA methylation changes in cancer ranging typically from the TSS ± 300 bp to TSS ± 2 kb (24,35). The promoters were then subset for those regulating autosomal protein-coding genes that also overlapped one or more probes from the Illumina Infinium 450K BeadChip array. Probes which bind to non-CpG sites, are for detection of SNPs, which were previously flagged as being cross-reactive (36), or were associated with a large number of missing values (≥10% of all tumour samples) in the TCGA methylation files were excluded. In addition, we also removed probes whose beta values had a Pearson’s correlation value of ≥0.4 with tumour purity in BRCA. We chose to use BRCA as it is the cancer type with the largest number of tumour samples in TCGA and used purity scores calculated using the InfiniumPurify R package (values downloaded from https://doi.org/10.5281/zenodo.253193) (37). We used Pearson correlation as we often observed strong linear relationships between tumour purity and probe methylation values. This resulted in a final set of 94 101 probes which overlap 23 208 promoters regulating 14 773 distinct genes, with 5956 genes having two or more different promoters (see Supplementary Table S1 for a complete list of promoter genomic coordinates and associated genes and Infinium 450K probes).

Enhancer regions for hg19 were downloaded from ENSEMBL using the biomaRt R package (38).

### TCGA promoter methylation

Illumina Human Methylation 450 files with probe beta values for TCGA tumour and normal samples were downloaded from the Genomic Data Commons (GDC) using the GDC-client tool. Promoter methylation values for each sample were calculated by taking the mean of beta values of the probes overlapping each promoter.

Thirteen cancer types had adequate matched tumour-normal pairs to test differential methylation: bladder urothelial cancer (BLCA, n = 21), breast invasive cancer (BRCA, n = 90), colon adenocarcinoma (COAD, n = 38), esophageal carcinoma (ESCA, n = 16), head and neck squamous cell carcinoma (HNSC, n = 50), liver hepatocellular carcinoma (LIHC, n = 50), lung adenocarcinoma (LUAD, n =29), lung squamous cell carcinoma (LUSC, n = 40), kidney renal clear cell carcinoma (KIRC, n = 160), kidney renal papillary cell carcinoma (KIRP, n = 45), prostate adenocarcinoma (PRAD, n = 50), thyroid carcinoma (THCA, n = 56), uterine corpus endometrial carcinoma (UCEC, n = 33).

### Epigenetic covariates for DNA methylation

BigWig files for 877 features, comprising 33 distinct epigenetic marks profiled across up to 111 different cell/tissue types, were downloaded from the Roadmap Epigenomics Project (39). Each of the 33 epigenetic marks is measured in between 1 and 111 of the different samples. For example, H3K27me3 and H3K4me3 are measured in all 111 samples, H3K27ac is measured in 82 samples, H3K79me2 in 5 samples and H3K79me2 in only 1 sample. As we consider the same epigenetic mark measured in different samples to be different features, we have 111 different features for H3K27me3 measured in different samples, 82 features for H3K27ac measured in different samples, 5 features for H3K79me2 measured in different samples, just one feature for H3K79me2 etc. The values of these features in promoters were calculated using the bigWigAverageOverBed tool from UCSC (40) and using the mean of covered bases only. In addition, we also calculated CpG density, the number of CpGs in a promoter divided by the promoter length, for each promoter and used this along with the Roadmap features, bringing our total number of features to 878.

### MethylDriver algorithm

In order to identify which promoters are most similar to each other epigenetically, principal component analysis (PCA) was performed on the full set of promoters using all of the 878 promoter features. For each individual promoter, which we call the home promoter, Euclidean distances to all the other promoters were calculated using the principal component values and used to determine a selected number of the most epigenetically similar promoters to the home promoter, which we refer to as the home promoter’s epigenetic neighbourhood. If any other promoters overlapped the genomic range of the home promoter, they were excluded from becoming part of it’s epigenetic neighbourhood.

For our analyses, we used epigenetic neighbourhoods of size 100. We also compared the results of using a range of different neighbourhood sizes and found that similarly sized neighbourhoods tend to give quite similar results (e.g. the putative drivers identified using neighbourhoods of size 100 and 200 had a Jaccard index of around 0.74). We found that a neighbourhood size of 200 gave the highest mean fold enrichment for TSGs across the different cancer types (3.9), with 100 a close second with a mean of 3.82. However, in terms of statistical significance, neighbourhood sizes of 25, 50 and 100 each had significant overlaps with 10 different cancer types, while larger neighbourhoods resulted in fewer cancer types with a significant overlap.

For overlap with unexpressed genes, a neighbourhood size of 400 had the lowest mean fold depletion with a mean depletion of 0.29. A size of 100 was similarly low with a mean depletion of 0.446. In terms of significant overlaps, a neighbourhood size of 25 resulted in significant overlap in 13/13 cancer types, with neighbourhoods of 50–2000 each resulting in 12 significant cancer types.

We chose to use a neighbourhood size of 100 as we felt if offered a good compromise between fold enrichment of these validation gene sets and statistical power.

For each cancer type, the mean difference in DNA methylation values between tumour and matched normal samples for each promoter were used to fit normal distributions to the epigenetic neighbourhood of each promoter, called its neighbourhood methylation change distribution. We also repeated our analyses fitting beta distributions instead of normal distributions and discovered that the resulting significant promoters were almost identical. We therefore decided to carry out all further analyses using the lists of promoters obtained by fitting normal distributions.

The significance of the mean methylation change value of a home promoter was calculated using the cumulative distribution function of its neighbourhood methylation change distribution and the resulting *P*-values were corrected by FDR. Promoters which differed significantly from their neighbourhood distribution and had a mean increase in methylation in tumours compared to matched normal samples were designated hypermethylated, while those that differed significantly from their neighbourhood distribution and had a mean decrease in methylation were designated hypomethylated.

We also tested MethylDriver using just Roadmap features from the tissue of origin for both BRCA and COAD (i.e. features from breast or colon samples, respectively) but did not see any improvement in performance.

### Comparison with other tools

The uncorrected sets of differentially methylated genes were identified using Wilcoxon signed-rank tests with the promoter methylation values from matching tumour-normal pairs and correcting for multiple testing using the Benjamini–Hochberg procedure.

MethylMix version 2.18.0 (25) was run by providing a table with the methylation values of promoters in tumour samples, a table with the promoter methylation values in matching normal samples and a table with the gene expression values in tumour samples for a given cancer type, with default settings for all other parameters. Since MethylMix requires tables with matching promoter methylation values and gene expression values, it was run choosing only one promoter for each gene.

Comb-p (27) was run as the following command ‘comb-p pipeline -c 4 –seed 0.05 –dist 750 -p $BED’, where $BED is a sorted BED file with the coordinates of all probes and a fourth column with the *P*-values from Wilcoxon signed-rank tests for the differences between the probe methylation values in tumour and normal samples. Promoters were then overlapped with the resulting significant regions to determine differentially methylated promoters.

Differentially methylated genes from DNMIVD (41) for TCGA were downloaded from http://119.3.41.228/dnmivd/download/, and differentially methylated genes for RESET were retrieved from Saghafinia et al (24). The top 500 differentially methylated genes for each cancer type from MethSig were retrieved from Pan *et al.* (35).

### Gene set definitions

In order to define the sets of differentially expressed genes TCGA RNA-seq HTSeq count files were downloaded using the GDC-client for each cancer type. The files were filtered to only include protein-coding genes and then analysis of differential expression between tumour and normal samples was performed using DESeq2 (42).

Normal tissue-specific unexpressed protein-coding genes were defined as genes which had a median TPM value of 0 and also a max TPM value <5 in the available matching normal samples for each cancer type. This resulted in an average of almost 1900 unexpressed genes per cancer type (see Supplementary Table S2 for full lists of unexpressed genes for each cancer type). It could be that many of the unexpressed genes may be associated with the small number of promoters whose methylation is positively associated with gene expression, making them unsuitable candidates for false positive hypermethylated driver genes. To rule out this possibility, we checked the overlaps of unexpressed genes and genes with promoters who have a Spearman correlation >0.1 with TPM of the associated gene. This was only the case for <5% of unexpressed genes.

GO term enrichment was performed using the ConsensusPathDB web tool (43), using all gene ontology levels of the biological process and molecular function domains. All unique genes from our promoter set were used as the background.

A total of 177 TSGs (https://www.uniprot.org/uniprot/?query=keyword:KW-0043) and 229 oncogenes (https://www.uniprot.org/uniprot/?query=keyword:%22Proto-oncogene%20[KW-0656]%22) were downloaded from Uniprot (9 September 2020), filtering for human protein-coding genes (see Supplementary Table S3 for full lists of oncogenes and TSGs). Paralogous gene pairs were downloaded using biomaRt. GO semantic similarity for gene pairs was calculated using the GOSemSim R package with Biological process GO terms (44).

### Statistical analyses

All statistical analyses were performed using R version 4.0.3.

## RESULTS

### Promoter methylation changes in cancer can be partially explained by epigenetic features in normal cells

**Figure 2.**
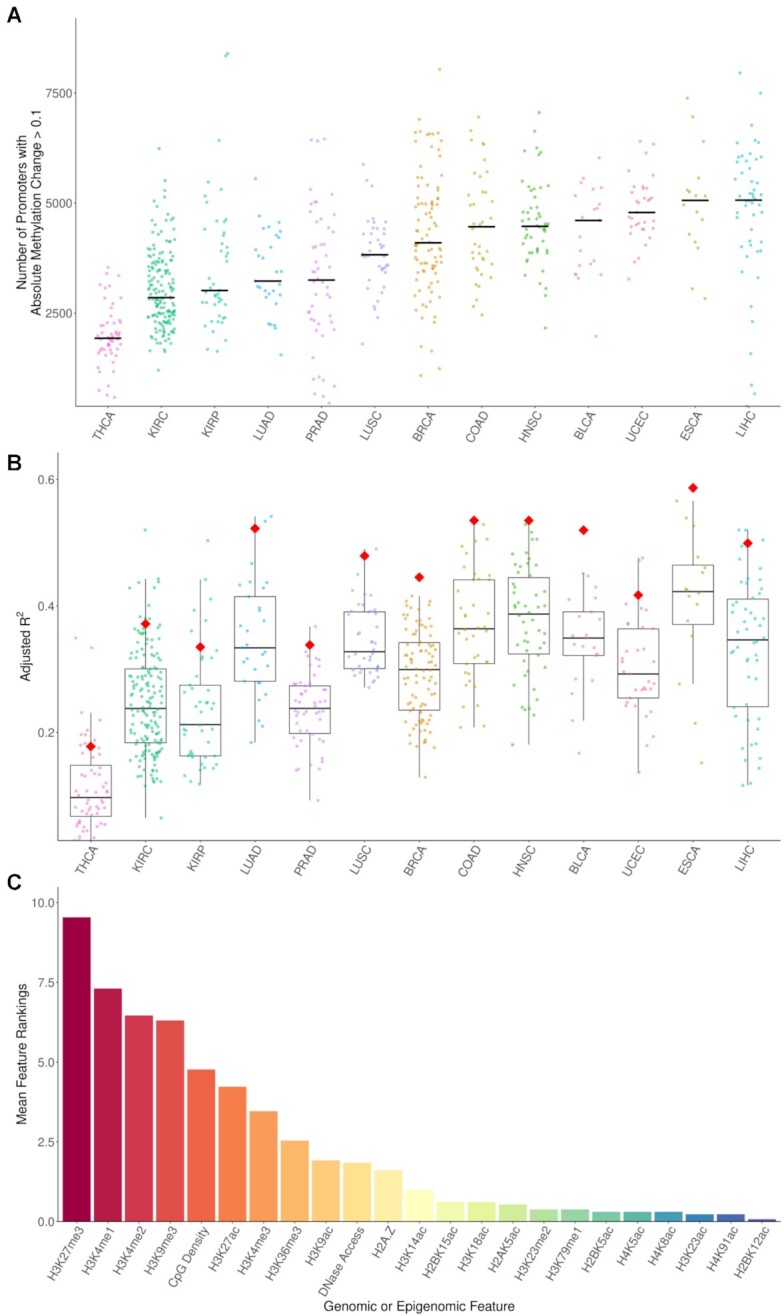
Methylation change and variance explained by Roadmap features. (**A**) The number of promoters per patient with an absolute methylation change >0.1. (**B**) The adjusted *R*^2^ of linear models fit to the promoter methylation change in individual TCGA samples using Roadmap promoter features. Red diamonds indicate the adjusted *R*^2^ of models fit to the consensus methylation change for each cancer type (the mean change value of each promoter in all patients with a given cancer type). Methylation changes in cancer types with greater numbers of promoters with methylation changes above 0.1 tend to be better explained by the Roadmap features. (**C**) ranking of the most important epigenetic marks/genomic properties for determining methylation change in cancer. A modified form of stepwise selection in which each epigenetic mark could only be added once (and thus excluding multiple features comprising the same epigenetic mark profiled in different cell types) was used to identify the 10 most epigenetic marks or genomic features for predicting methylation change in each cancer type. To identify which marks were the most important across the models fit to the different cancer types, we performed a type of positional voting in which features received a number of points based on how important they were to each model. The most important feature for a cancer type was given a score of 10 points, the second most important feature a score of 9 points and so on, until the 10th most important feature, which was given a single point. The sum of the ranking scores divided by the number of models, 13, gave the mean feature ranking for a mark.

Promoter methylation changes occur frequently in cancer, with samples typically exhibiting thousands of promoters with altered methylation (Figure 2A), fitting similar observations made by others before (24). DNA methylation changes in cancer have previously been shown to be closely associated with properties of the genome and epigenome, including trimethylation of H3K27me3 (45–47), as well as replication timing and chromatin accessibility (31–33). We thus postulate that many of these events may be passenger events whose methylation change is due to their genomic location and associated epigenetic properties rather than their role in tumorigenesis. We first investigated which genomic and epigenomic features in normal cells correlate with DNA methylation changes at promoter elements in cancer, using data from the Roadmap Epigenomics Project (39). Supplementary Figure S1 displays the correlation values for the different chromatin features and promoter methylation change in COAD.

To quantify how much variation in tumour promoter methylation change we can explain with the complete set of epigenetic features, we fit multivariable linear models to the methylation change in each patient. We observed that we can explain a substantial proportion of the variation (adjusted *R*^2^ 95% confidence interval = 0.3 ± 0.01). The proportion of variance explained differs across tumour types and also between patients with the same tumour type, with a median adjusted *R*^2^ of over 0.4 for patients with both ESCA and PAAD. In contrast, THCA had a median adjusted *R*^2^ of only 0.1 (Figure 2B). While the relationships between DNA methylation change and levels of epigenetic marks tended to be non-linear (see scatter plots in Figure 1), we used linear models simply for their ease of interpretation.

Interestingly, we observed that the promoters which are predicted by the multivariable linear models to undergo the greatest methylation change in cancer (either gain or loss) have substantially higher variation in methylation levels in normal samples than promoters predicted to experience little methylation change in cancer (Supplementary Figure S2).

We used a modified form of forward stepwise selection to identify the 10 most important epigenetic marks or genomic features for predicting DNA methylation change in each cancer type. As we treat the same epigenetic mark profiled in different samples as separate features, we can have many different features related to the same epigenetic mark. For example, H3K27me3 is profiled in 111 different samples in the Roadmap Epigenomics project and thus we have 111 different H3K27me3 features. We modified traditional feature selection to ensure that only one feature could be selected for each epigenetic mark. Thus, if H3K27me3 levels in liver is identified as the most predictive feature, all other H3K27me3 features will be excluded when searching for the successive most useful features. We excluded WGBS features from selection as the relationship between DNA methylation levels in normal cells and methylation change in cancer should be obvious, with highly methylated genomic regions in normal cells exhibiting a tendency to gain methylation in cancer and lowly methylated regions exhibiting a tendency to gain methylation. We created a meta-ranking for features by combining their ranking in individual cancer types (Figure 2C).

H3K27me3 was the most predictive mark overall, being the top mark in 10 out of 13 cancer types and fitting with observations made previously. We also identified methylation of H3K4 as being highly predictive of DNA methylation change in cancer, especially mono- or dimethylation of H3K4, both of which had greater predictive power than H3K4me3. H3K9me3 and H3K27ac were also among the top features. CpG density was surprisingly the 5th most important feature overall, ranking ahead of the vast majority of epigenetic marks studied by the Roadmap Epigenomics project.

Interestingly, we noticed that the top predictive features for each mark were often from embryonic stem cells or fetal tissues, supporting the idea that DNA methylation change in cancer represents a reversion to an embryonic-like state. For example, H3K4me2 profiled in embryonic stem cells (ESCs) was the top feature overall in our modified feature selection when also taking sample type into account. H2A.Z in ESCs was also one of the top features overall. Furthermore, H3K9me3 in fetal neutrophils was the most predictive feature among the H3K9me3 features.

Next, we averaged promoter methylation changes within a cancer type to produce consensus methylation change values for each promoter for each cancer type and subsequently found that the epigenetic features could generally explain the variance in these cancer consensuses much better than for individual samples (Figure 2B, red diamonds), likely reflecting the reduction in noise in the cancer consensuses compared to individual samples.

Due to both the large number of differentially methylated promoters in tumours and the ability to explain much of the variation in tumour methylation change with the epigenetic features of normal cells and inspired by the MutSigCV method of Lawrence *et al.* (30), we developed MethylDriver (https://github.com/rheery/MethylDriver), a novel method which accounts for this covariance to identify promoters whose methylation change differs substantially from what would be expected given their epigenetic profile in healthy cells (Materials and Methods and Figure 1).

We found an increase compared to what would be expected by chance within the promoter neighbourhoods in the mean number of paralogous gene pairs (median of 4 pairs in the promoter neighbourhoods compared to 2 pairs in randomly generated neighbourhoods; *P*-value < 2.2 × 10^–16^). We also found a small but significant mean pairwise GO semantic similarity of genes (median of 0.21) for promoter neighbourhoods compared to a median of 0.20 for randomly generated neighbourhoods; *P*-value = 2.7 × 10^–14^). We also found that many of the identified promoter neighbourhoods exhibited an increased tendency to reside on the same chromosome as their home promoter (4988 neighbourhoods with *P*-value < 0.05 for an upper-tailed binomial test) and that the promoters on the home promoter’s chromosome tended to be closer than would be expected by chance (Wilcoxon rank-sum test *P*-value < 2.2e-16) with a median distance of 31 MB for the neighbourhood promoters compared to 37 MB for randomly selected promoters from the same chromosome.

To explore if the ability of epigenetic features in normal cells to explain methylation change in cancer is specific to promoters, we created random regions which did not overlap promoters but resembled promoters in terms of their CpG density and investigated how well we could explain their mean methylation change in three pairs of matched tumour-normal prostate cancer samples with WGBS data from the CPGEA project. We found that we could explain a similar proportion of the variation (adjusted *R*^2^ of 0.45) as with promoter methylation change and that marks like H3K27me3, H3K4me1 and H3K27ac were also positively correlated with methylation change in cancer in these random regions.

The methylation changes at enhancers in cancer have received much less attention than promoters, though they are increasingly being implicated in carcinogenesis (48). We investigated how much of the variation in mean enhancer methylation in the same three pairs of matched tumour-normal prostate cancer samples we could explain using the same set of epigenetic features as for the promoters. We found that the corresponding multivariable linear model had an adjusted *R*^2^ of 0.27, indicating that methylation change at enhancers in cancer can also be partially explained by the presence of epigenetic marks in normal cells, though additional features which explain methylation change at enhancers may remain to be identified.

### Correcting for epigenetic covariates results in a smaller number of promoters with greater biological relevance of differentially methylated genes

We applied MethylDriver to the promoter methylation difference between matched tumour and normal samples across 13 different cancer types. Several tools have previously been developed to determine differentially methylated regions between cohorts or conditions but few of them take genomic or epigenomic covariates of DNA methylation change into account and it is not clear which tools perform best when it comes to detecting biologically relevant DNA methylation changes in cancer.

We decided to compare the differentially methylated genes identified by MethylDriver to five other tools: one generic tool based on its superior performance in a benchmarking study (49) (comb-p (27)) and four based on their particular relevance to identify differentially methylated genes in cancer (RESET (24), MethylMix (25) DNMIVD (41) and MethSig (35)). RESET and MethylMix incorporate expression data as an additional filter criterion and thereby control for the large number of otherwise differentially methylated genes. DNMIVD uses a stringent cutoff on the beta value fold change in addition to a statistical cutoff to control result set size. We also evaluated the genes detected as significant by simply applying a Wilcoxon rank-sum test, which is representative of the most naive statistical attempt to detect differentially methylated genes and which we henceforth refer to as the uncorrected approach.

**Figure 3.**
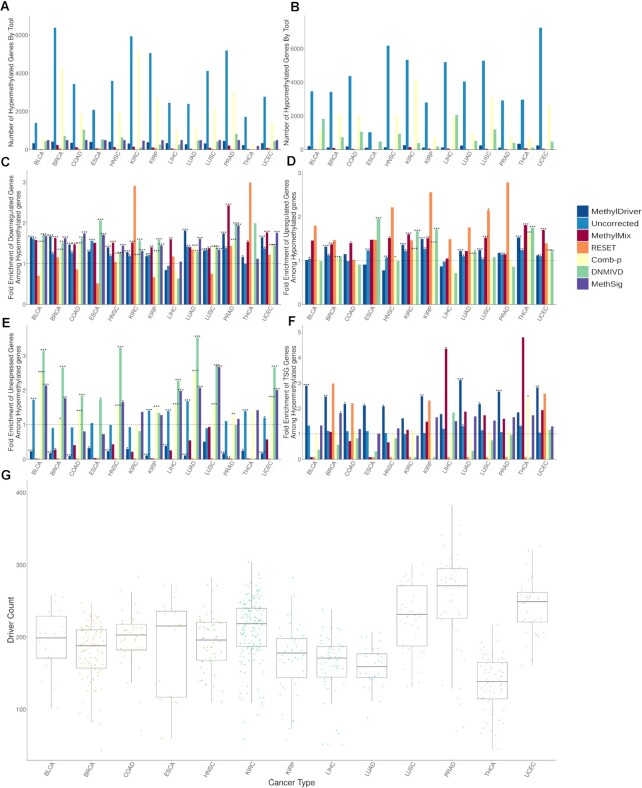
Correction for covariates increases the overlap with relevant gene sets and eliminates likely spurious associations. (**A** and **B**) Numbers of hypermethylated and hypomethylated genes of different tools. (**C** and **D**) Enrichment of downregulated genes among hypermethylated genes and enrichment of upregulated genes among hypomethylated genes. (**E**) Enrichment of TSGs among hypermethylated genes. (**F**) Enrichment of unexpressed genes among hypermethylated genes. (**G**) Number of putative methylation driver events in samples across different cancer types. Significance calculated using Chi-squared test (panesl C and D) or Fisher’s exact test (panels E and F): * indicates *P*-value < 0.05, ** *P*-value < 0.01 and *** *P*-value < 0.001.

The different tools resulted in hugely differing numbers of hypermethylated and hypomethylated genes, with both the uncorrected approach and comb-p detecting several thousand differentially methylated genes while other tools identified <100 genes per cancer type. MethylDriver identified an average of ∼350 hypermethylated genes and an average of ∼200 hypomethylated genes (Figure 3A and B). See Supplementary Tables S4 and S5 for lists of hypermethylated and hypomethylated genes identified by MethylDriver in each TCGA cancer type studied.

MethylDriver should remove many false positive differentially methylated drivers and result in increased biological relevance of the identified differentially methylated genes. Thus, we compared the overlap of differentially methylated genes identified by MethylDriver and those identified by the other tools and the uncorrected sets with three different categories of genes relevant to cancer: differentially expressed genes, unexpressed genes and TSGs.

It should be expected that there is a high overlap of hypermethylated genes with downregulated genes and, likewise, for hypomethylated genes and upregulated genes. We compared the numbers of downregulated genes that overlapped the sets of differentially methylated genes identified by MethylDriver and the other tools. Differentially methylated genes identified by MethylDriver had a significant overlap with both downregulated genes (for hypermethylated genes) and upregulated genes (for hypomethylated genes), performing as well as or better than other tools in most cancer types.

Conversely, it should be expected that there are low overlaps with the reverse associations: hypermethylated genes with upregulated genes and hypomethylated genes with downregulated genes. We also compared these overlaps and we saw lower overlaps than would be expected by chance with the gene sets resulting from most tools (Supplementary Figure S3).

In a supporting analysis, given that the association of promoter methylation values and expression of the corresponding gene has been reported to vary widely (50), we investigated the correlations between promoter methylation values and TPM values of the corresponding genes separately in different cancer types using tumour samples from TCGA. There was a median correlation of -0.06 (Spearman’s rho) between promoter methylation level and TPM values for the corresponding gene, although there was large variation in the correlation values, with some relatively strong negative correlations and even a small number of relatively strong positive correlations (Supplementary Figure S4), fitting with previous observations (50,51).

We next asked if the differentially methylated promoters detected by MethylDriver displayed stronger associations between their methylation and transcriptional expression of the corresponding gene compared to the average associations for all promoters. We therefore compared the distribution of methylation-TPM Spearman correlations of the differentially methylated promoters detected by MethylDriver to those of all promoters. We observed a highly significant difference in the mean correlation values (*P*-value < 2.2e-16, lower-tailed Wilcoxon rank sum test), with a median correlation value of -0.15 for the differentially methylated promoters detected by MethylDriver, more than twice as strong as the average association for all promoters (Supplementary Figure S4). Furthermore, only 20% of the methylation-TPM correlations were under -0.2 for all promoters, while this proportion increased to over 40% for MethylDriver promoters.

As hypermethylation has been found to occur in tumours even at the promoters of genes unexpressed or lowly expressed in the corresponding normal tissue (52–54), these seemed like a good choice for false positives drivers for hypermethylated genes. As hypermethylation of gene promoters is generally associated with transcriptional silencing, there should be little biological consequence of methylating a gene which is already unexpressed and thus they should be underrepresented among epigenetic drivers in cancer. Thus, we compared the overlap of hypermethylated genes identified by the different tools for each cancer type with genes unexpressed in the corresponding normal tissue. We found a statistically significant enrichment of unexpressed genes among the uncorrected set for most cancer types and also for comb-p, DNMIVD and MethSig (*P* < 0.05 with two-sided Fisher’s exact test). In contrast, we observed a significant depletion of unexpressed genes in MethylDriver hypermethylated genes for almost all cancer types (Figure 3E).

Since TSGs are well-established to be frequently silenced by hypermethylation in cancer, they seemed an obvious choice for true positives for hypermethylated genes (13–15). We contrasted the overlap of hypermethylated genes from the different tools with TSGs. With MethylDriver, there was significant enrichment in TSGs for the hypermethylated genes in the majority of cancer types (*P*-value < 0.05 with Fisher’s exact test; Figure 3F). In contrast, there was no significant enrichment in TSGs for all other tools in most cancer types.

With hypomethylated genes and oncogenes, there was generally no enrichment for any tools, including MethylDriver (Supplementary Figure S5), suggesting that differential methylation in cancer tends to favour silencing of TSGs rather than de-repression of oncogenes. Together, the reduced overlaps with unexpressed genes and the increased overlaps with TSGs strongly suggest greater biological relevance of the corrected hypermethylated genes.

Given that different cancers are known to have different number of commonly mutated driver genes (55), we were curious if the number of putative methylation driver events similarly varied with tumour site. We calculated the overlap of putative methylation driver events detected when applying MethylDriver to individual samples within a cancer type with those detected when using the methylation change consensus. We found great variety within each tumour type, but also noted that some cancers tend to acquire greater numbers of putative methylation driver events than others. Interestingly PRAD has relatively few frequently mutated driver genes (55), with *SPOP* being the only gene mutated in over 10% of samples (56), yet PRAD tends to acquire more putative methylation driver events than any other cancer (Figure 3G).

### Functions of differentially methylated genes in cancer

**Figure 4.**
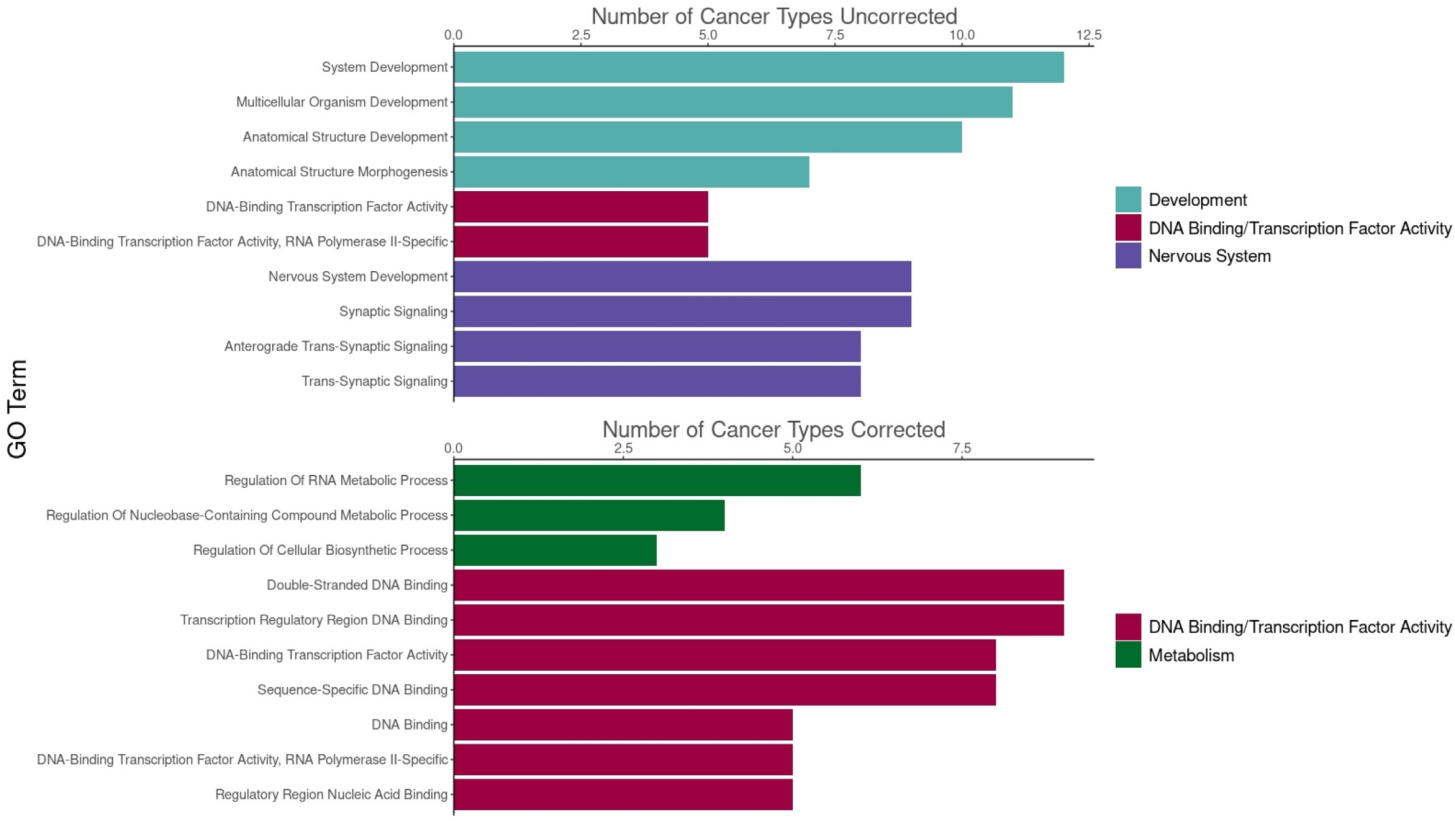
Functions of differentially methylated genes in cancer. Enriched GO terms in hypermethylated corrected and uncorrected genes. For each cancer type, the top 10 most significant terms were selected (or all terms if there were ≤10 significant terms using a *q*-value cutoff of 0.1). Then the frequency of these top-ranking terms were compared between corrected and uncorrected sets. Barplots display the top 10 most frequent terms for without and with correction.

After having established that the corrected sets of differentially methylated genes appeared to display increased relevance to cancer in terms of overlap with differentially expressed genes and TSGs, we sought to investigate which roles these genes may be playing during tumorigeneis. GO term enrichment analysis revealed distinct differences in the terms that were most significant in the uncorrected and corrected hypermethylated gene sets, with the most significant uncorrected terms consisting of mostly nervous system- and development-related GO terms (Figure 4). The enrichment of developmental genes in the uncorrected gene sets likely reflects the association between DNA methylation change and H3K27me3, which plays a crucial role during development, and hence the DNA methylation change in cancer for many of these genes may just be a consequence of high levels of H3K27me3 at their promoters.

To understand the impact of correcting for the epigenetic covariates on the functional composition of differentially methylated genes, we repeated the functional enrichment analysis after running MethylDriver. The top terms from the uncorrected set were composed of terms related to metabolism, DNA replication and transcription factor activity (Figure 4). For hypomethylated genes, the most enriched terms were related to immune system regulation for both uncorrected and corrected sets (Supplementary Figure S6).

We noticed that genes encoding chromatin modifiers were often hypermethylated in several cancer types, including the histone methyltransferases *KMT2C* (11 cancer types) and *NSD1* (7 cancer types) and the histone demethylase *KDM2B* (6 cancer types). Indeed, the hypermethylated genes from BLCA, KIRC, KIRP and THCA had significant overlaps with the Reactome pathway chromatin modifying enzymes (Fisher’s exact test, FDR-corrected *P*-value < 0.05). Of particular interest were the hypermethylation of *DNMT3A*, a DNA methylation writer, in five cancer types and *TET2*, a DNA methylation eraser, in four cancer types, suggesting a possible mechanism in which global DNA methylation changes may be effected via hypermethylation of these targets.

There were several chromatin modifiers also among the hypomethylated genes, including the histone acetyltransferase *NCOA1* in four cancer types, the histone deacetylase HDAC9 in two cancer types, and the histone demethylases KDM1A, KDM2A, KDM4A, KDM6B and the histone methyltransferase KMT5C in one cancer type each.

### Glutathione S-Transferase promoter methylation in prostate cancer

We noted that terms related to glutathione metabolism (glutathione peroxidase activity and glutathione binding, both *q*-value < 0.1) were among the most significantly enriched GO terms among the corrected hypermethylated genes in prostate cancer (Supplementary Table S6), and also that Glutathione S-transferase (GST) family members *GSTM1*, *GSTM2* and *GSTP1* were among the corrected hypermethylated genes in prostate cancer. As GSTs appear to protect against DNA damage (57,58), we wondered if there is a relationship between promoter methylation of GSTs and the tumour mutation burden (TMB) in prostate cancer. Indeed, we uncovered a clear association between promoter methylation and overall TMB for *GSTM2* and *GSTP1* (Spearman’s ρ of 0.43 for both *GSTM2* and *GSTP1*; Supplementary Figure S7A and B) but not for *GSTM1*. Both *GSTM2* and *GSTP1* promoters were among the top 1% of promoters whose methylation is most strongly correlated with TMB in prostate cancer (Supplementary Figure S7C) demonstrating that this association is specific for these genes. Similarly, we also found strong correlations of *GSTM2* and *GSTP1* promoter methylation with copy number alteration (CNA) burden in prostate cancer (Spearman’s ρ of 0.53 for GSTM2 and 0.49 for *GSTP1*; Supplementary Figure S7D and E), with both promoters among the promoters whose methylation is most strongly associated with CNA burden (Supplementary Figure S7F). Together these observations suggest a general role for *GSTM2* and *GSTP1* in controlling genomic integrity, which is counter selected in prostate cancer.

Implying a causal role for *GSTM2* and *GSTP1* promoter methylation in increased TMB and CNA, promoter methylation was observed to be anti-correlated with transcriptional expression of both genes and in turn their transcriptional expression was found to be anti-correlated with TMB and CNA (Supplementary Figure S8). To establish a direct link between *GSTM1* and *GSTM2* activity and tumor growth rate, we tested if their promoter methylation status is associated with the expression of the marker of proliferation Ki-67. Indeed we identified a positive association (Spearman’s ρ of 0.29 for *GSTM2* and 0.28 for *GSTP1*; Supplementary Figure S7G and H) and once again they were among the promoters with the highest correlation values (Supplementary Figure S7I).

Thus, we reasoned that hypermethylation of *GSTP1* and *GSTM2* may be associated with increased probability of acquiring driver gene mutations in prostate. We tested the top 10 most frequently mutated genes in PRAD and found that both *TP53* and *FOXA1* displayed increased mutation frequencies in the top quartile of samples ranked by GSTP1 promoter methylation compared to the bottom quartile (FOXA1 *P*-value = 0.0002; TP53 *P*-value = 0.026; both Fisher’s exact test). We saw no association of *GSTM2* methylation and mutation of individual genes.

**Figure 5.**
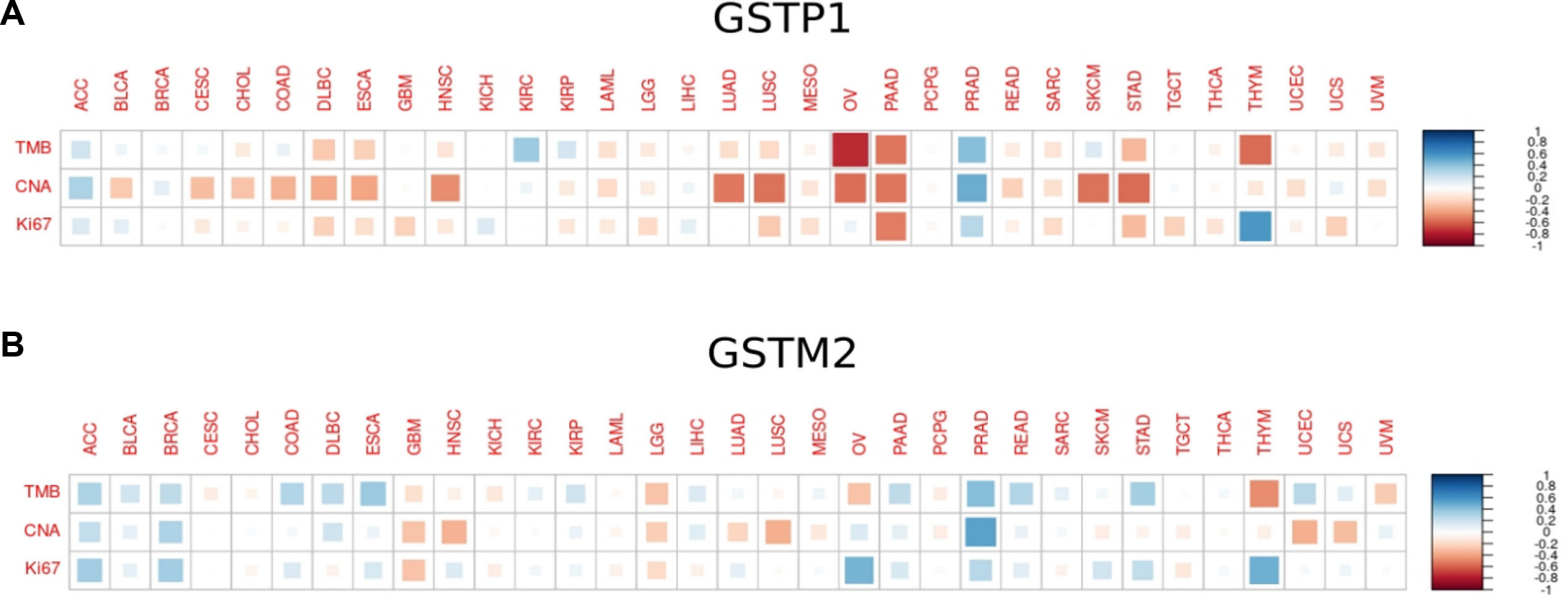
Correlation plots of GSTP1 (**A**) and GSTM2 (**B**) Promoter Methylation with TMB and CNA burden and Ki-67 expression. Colour indicates Spearman correlation values.

We then investigated the association of *GSTP1* and *GSTM2* promoter methylation with the TMB and CNA burdens and expression of the proliferation marker Ki-67 in all other cancer types from TCGA. We were surprised to discover that *GSTP1* methylation displays a unique pattern in PRAD compared to all other cancers, with relatively strong positive associations for all three, in contrast to most other cancer types which display very weak or even negative correlations, indicating that *GSTP1* may play a unique role in protecting the prostate from mutations (Figure 5).

Since a large number of matched normal-tumour samples from Asian prostate cancer patients has recently been analysed with whole genome bisulfite sequencing (59) as part of the CPGEA project, we were curious if GST family members are also hypermethylated in this cohort, in addition to the western TCGA cohort. We applied MethylDriver to this dataset and found that both *GSTM2* and *GSTP1* were again hypermethylated, with several glutathione-related GO terms being over-represented (glutathione binding, glutathione peroxidase activity and glutathione derivative metabolic process; all *q*-value < 0.1). Thus, hypermethylation of GST gene promoters seems to be a universal feature of prostate cancer.

### Recurrence of hypermethylated genes across cancer types

**Figure 6.**
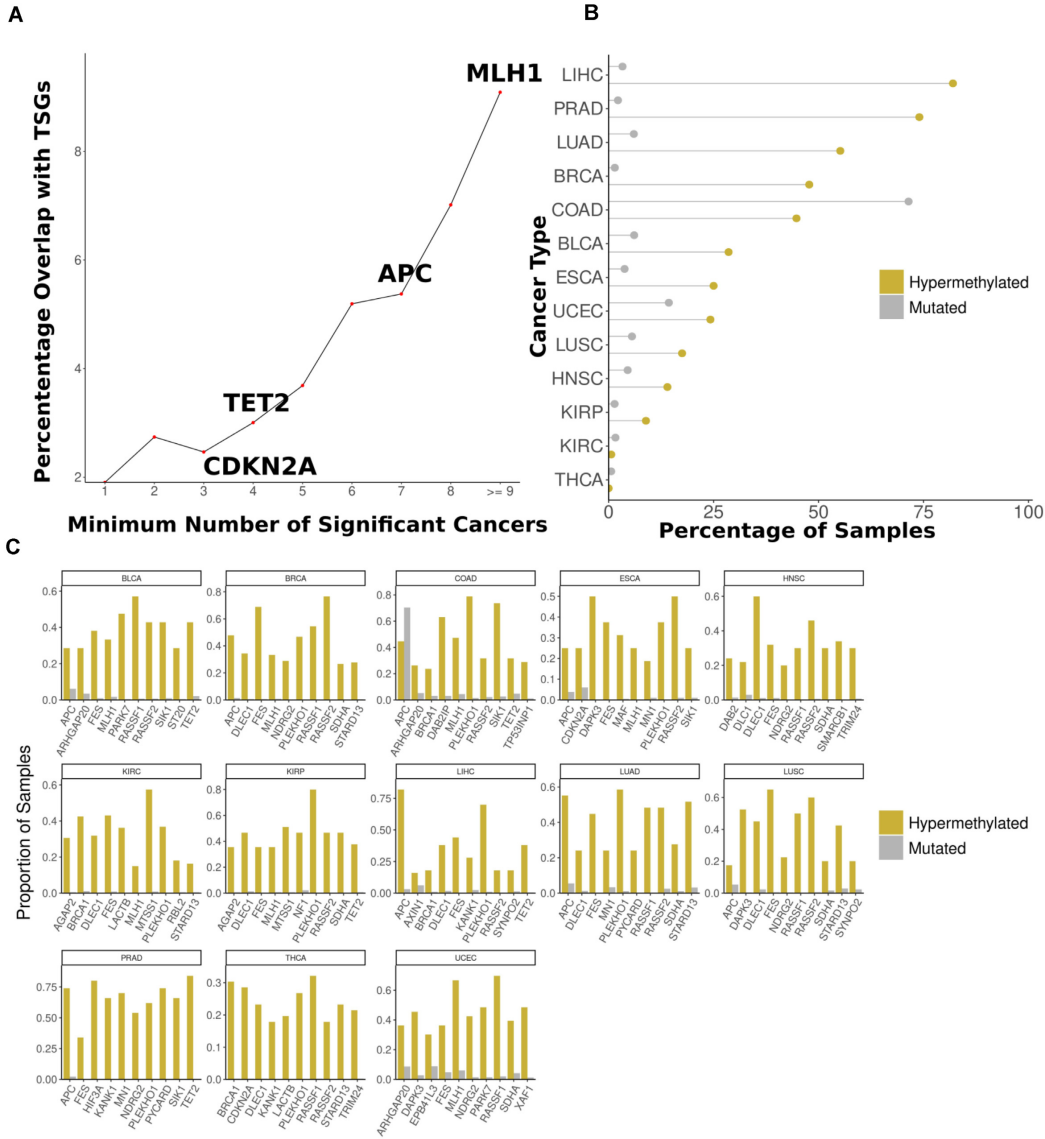
Hypermethylation of some TSGs is recurrent across different cancer types. (**A**) Overlap with TSGs increases in genes hypermethylated in a greater number of cancer types. Selected TSGs hypermethylated in the different numbers of cancer types are shown. (**B**) Comparison of the proportion of samples where APC is mutated and hypermethylated. (**C**) Frequency of hypermethylation compared to frequency of mutation of the top 10 most frequently methylated TSGs in each cancer type.

We noticed that often genes are hypermethylated in several cancer types and that after creating groups of hypermethylated genes by the minimum number of cancer types in which they are hypermethylated (e.g. genes that are hypermethylated in at least one cancer type, genes that are hypermethylated in at least two cancer types and so on) that the overlap with TSGs increases with groups of genes methylated in more cancer types (Figure 6A).

As already mentioned for DNA repair genes, distinct sets of genes have been reported to be inactivated through mutation and promoter hypermethylation. We noted that while *APC* is only mutated in a substantial proportion of COAD samples, it is significantly hypermethylated in COAD along with several other cancer types (BLCA, BRCA, LIHC, LUAD, PRAD and UCEC), being hypermethylated in 50% of samples or more in several tumour types (Figure 6B). We also made similar observations for numerous other TSGs, particularly MLH1, TET2 and CDKN2A, with them often being hypermethylated much more frequently than mutated in numerous cancer types (Figure 6C). Thus, some tumours seem to display preferences for inactivating certain genes through promoter hypermethylation rather than mutation, as has been previously described (60).

## DISCUSSION

Systematic studies of cancer genomes have identified a variety of mutated driver genes in the major cancer types (61–63). Many studies have taken various approaches to achieve something similar for differentially methylated genes in cancer and have uncovered novel roles for differentially methylated genes in tumorigenesis (24,32,64). These efforts typically identified many promoters that display aberrant methylation in a given tumour type, though the biological relevance of most of their associated genes to cancer is unclear and many are altered in a manner largely predictable from their epigenetic features in normal cells. Indeed, the changes at many promoters seem to reflect the activation of certain pre-programmed transcriptional and developmental processes in cancer development (65) rather than the individual role of their associated genes in tumorigenesis.

Recent approaches in cancer genomics take account of covariates that locally alter mutation frequency, for example chromatin accessibility (30,55). We are, to our knowledge, the first to apply this concept to epigenetic alterations by taking into account a diverse set of epigenetic covariates. We developed a new computational tool, MethylDriver, that can detect promoters displaying methylation changes in cancer substantially different from the expected background given a promoter’s epigenetic properties in normal cells and thus putatively causally involved in driving tumorigenesis.

We applied MethylDriver to 13 different cancer types and identified several hundred differentially methylated promoters in each type, generally less than one-tenth of the number of promoters that have significant changes without taking account of their epigenetic background. Only a small number (typically <10) of the dozens or even hundreds of non-synonymous somatic mutations a solid tumour harbors are thought to be driver mutations (66). We noted a similar pattern with DNA methylation events, with only ∼5% of differentially methylated genes in a cancer type without correction identified as putative drivers after we applied MethylDriver to individual samples in that cancer. Moreover, we found that the number of putative epigenetic driver events varies within and across tumour types, with PRAD having a particularly large number of putative epigenetic driver events on average.

Rather than only removing false-positive DNA methylation events, we would like to highlight that our method also detects events which would not be deemed significant with conventional statistical methods, such as the Wilcoxon signed-rank test. This occurs when there is only a small DNA methylation change between tumor and normal tissue, when the variation in this change is large or when there are only a small number of samples. On average, 10% of the differentially methylated promoters detected by MethylDriver in a cancer are not present in the corresponding uncorrected set. This reflects that MethylDriver can detect small methylation changes which are actually relatively large compared to the expected change given their epigenetic background. This includes promoters for TSGs, such as MLH1 and BRCA1, which in some cancer types were significantly hypermethylated after using MethylDriver but not by testing without correction.

Applying MethylDriver to several cancer types, we have identified a wider role for TSGs in cancer than just in the cancer types in which they are frequently mutated, with certain TSGs that are mutated specifically in a small number of cancer types, like APC in colorectal cancer, being hypermethylated in many more. Additionally, we have uncovered that putative epigenetic driver events, such as GSTP1 and GSTM2 hypermethylation in prostate cancer, may influence the acquisition of genetic driver events via their influence on the tumour mutation load.

As the accumulation of deleterious mutations should adversely impact the fitness of a tumour (67), we were intrigued why an increased mutation rate is apparently selected for in prostate cancer via hypermethylation of *GSTP1* and *GSTM2*. Indeed, CNA burden has been previously shown to be a strong predictor of recurrence and survival in prostate cancer (68,69) implicating methylation of *GSTM2* and *GSTP1* in the prognosis of prostate cancer patients via allowing for higher amount of genomic aberrations. The selection towards silencing genes involved in maintaining genome integrity may be partially explained by the relatively low mutation rate of prostate cancer compared to other cancers (30) and the small number of driver genes mutated frequently in prostate cancer (56). Thus, selection may favour an increased mutation rate in prostate cancer to increase the chances of inactivating one of the few prostate-specific driver genes.

There are most certainly other factors contributing to promoter methylation change in cancer besides those that we have identified, both in a general and tissue-specific manner, and their incorporation could lead to further improvement in our approach.

In summary, the correction for epigenetic covariates leads to identification of a set of differentially methylated putative cancer driver genes that potentially play diverse roles in tumour biology. Experimental validation of targets should be performed to definitively prove the biological role of identified genes in tumorigenesis. It also highlights an important link between accumulation of epigenetic changes and genomic mutations: in prostate cancer the silencing by DNA methylation of particular genes might make tumor cells more permissive for acquiring mutations. Finally, our work demonstrates a far more cancer type-general role for several tumor suppressors that previously have been associated with a small number of cancer types only. Taken together, this study expands our understanding of the epigenetic contribution to carcinogenesis.

## DATA AVAILABILITY

MethylDriver is available at https://github.com/rheery/MethylDriver.

All data from TCGA is available from GDC (https://portal.gdc.cancer.gov/repository) and the Asian prostate cancer WGBS data from CPGEA (https://wangftp.wustl.edu/∼hlee/SMMU/PC/WGBS_bedGraph/). For the analyses where we used only three samples from CPGEA, we used the tumour samples T1, T6 and T10 and their matching normal samples. The Roadmap Epigenomics data are available from https://egg2.wustl.edu/roadmap/data/byFileType/signal/consolidated/macs2signal/foldChange/. Transcription start sites for promoters were obtained from EPD (ftp://ccg.epfl.ch/epdnew/H_sapiens/006/Hs_EPDnew_006_hg19.bed).

## Supplementary Material

gkab1167_Supplemental_Files
