# Supplementary material for: DNA methylation variation along the cancer epigenome and the identification of novel epigenetic driver events
Source: Nucleic Acids Res. 2021 Dec 6;49(22):12692–705. doi: 10.1093/nar/gkab1167 (PMC8682778; doi:10.1093/nar/gkab1167)
Supplement: gkab1167_Supplemental_Files [file gkab1167_supplemental_files.zip › Supplementary Figures.pdf]

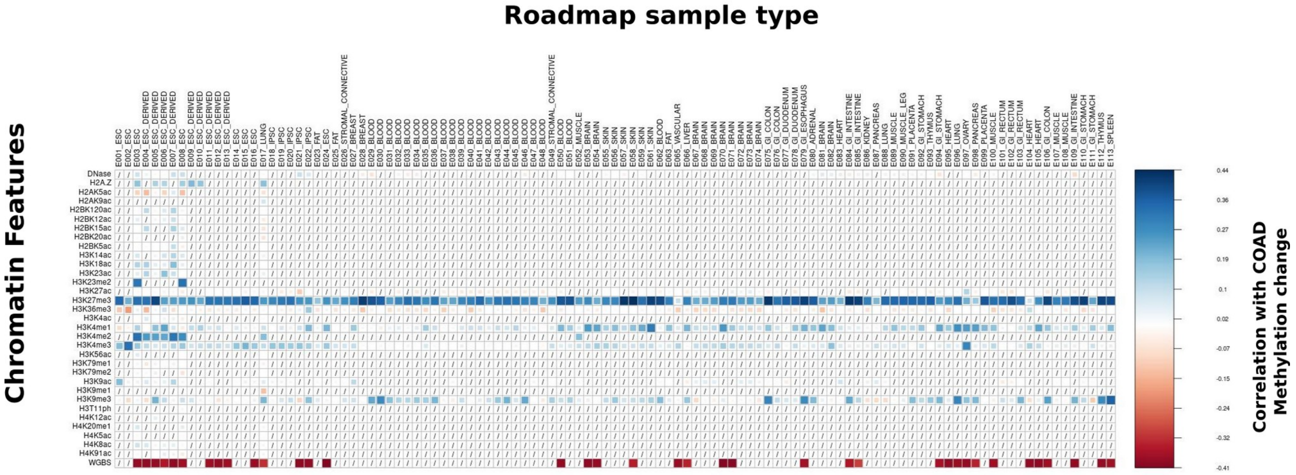

Supplementary Figure S1: Pearson correlation plot of promoter methylation change in COAD with chromatin features at promoters in different cell types from the Roadmap Epigenomics project. Horizontal label indicates the chromatin mark and vertical label indicates the sample ID and anatomy type of the sample from Roadmap with the colour indicating the correlation of the chromatin feature in the specified sample with the consensus methylation change observed at promoters in COAD. “/” indicates that a chromatin mark was not profiled in the specified sample.

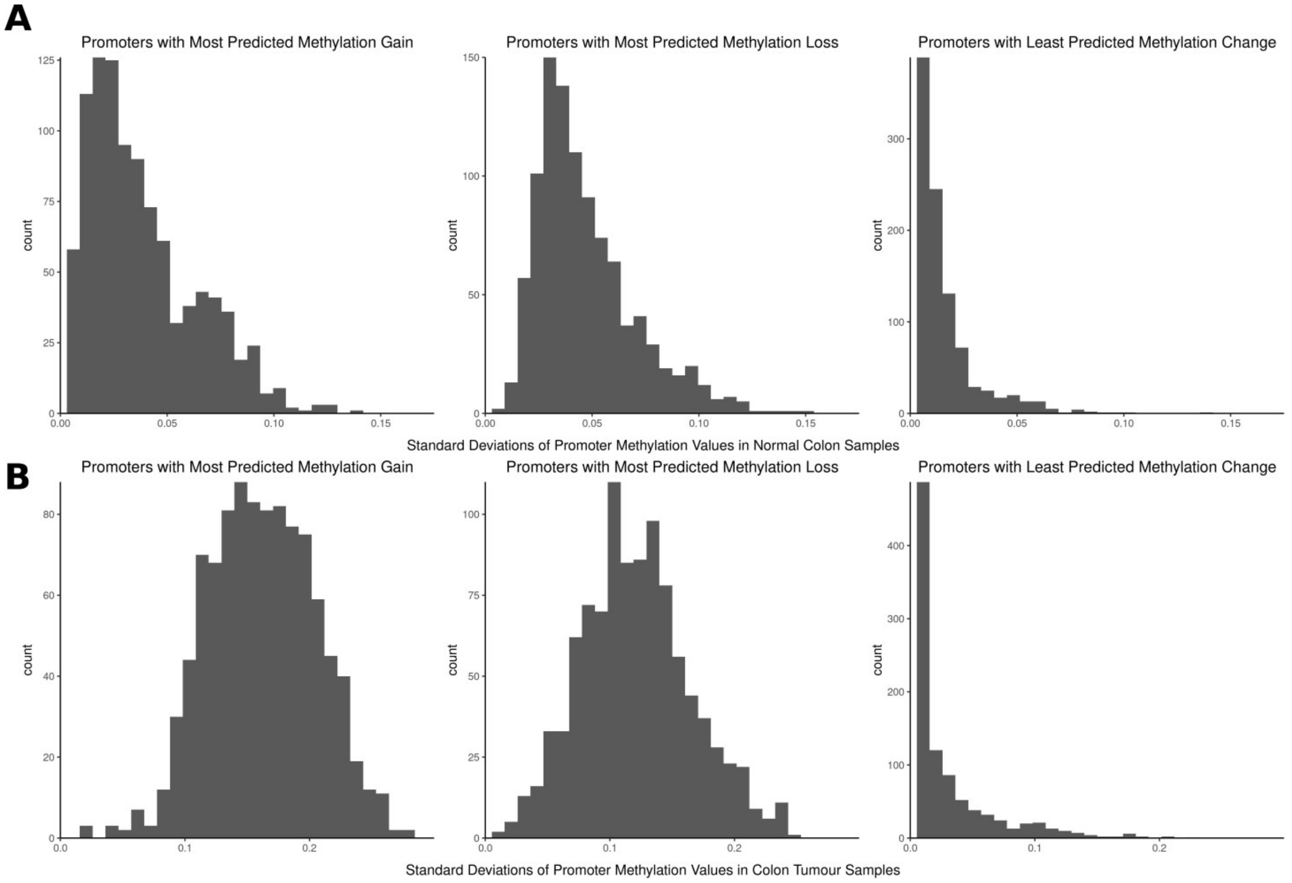

Supplementary Figure S2: Standard deviations of the methylation values of promoters in normal colon (A) and tumour colon samples (B) for the 1000 promoters with the greatest predicted methylation gain in COAD from multivariable linear models using epigenetic covariates, the 1000 promoters with the greatest predicted methylation loss in COAD and the 1000 promoters predicted to change methylation the least in COAD.

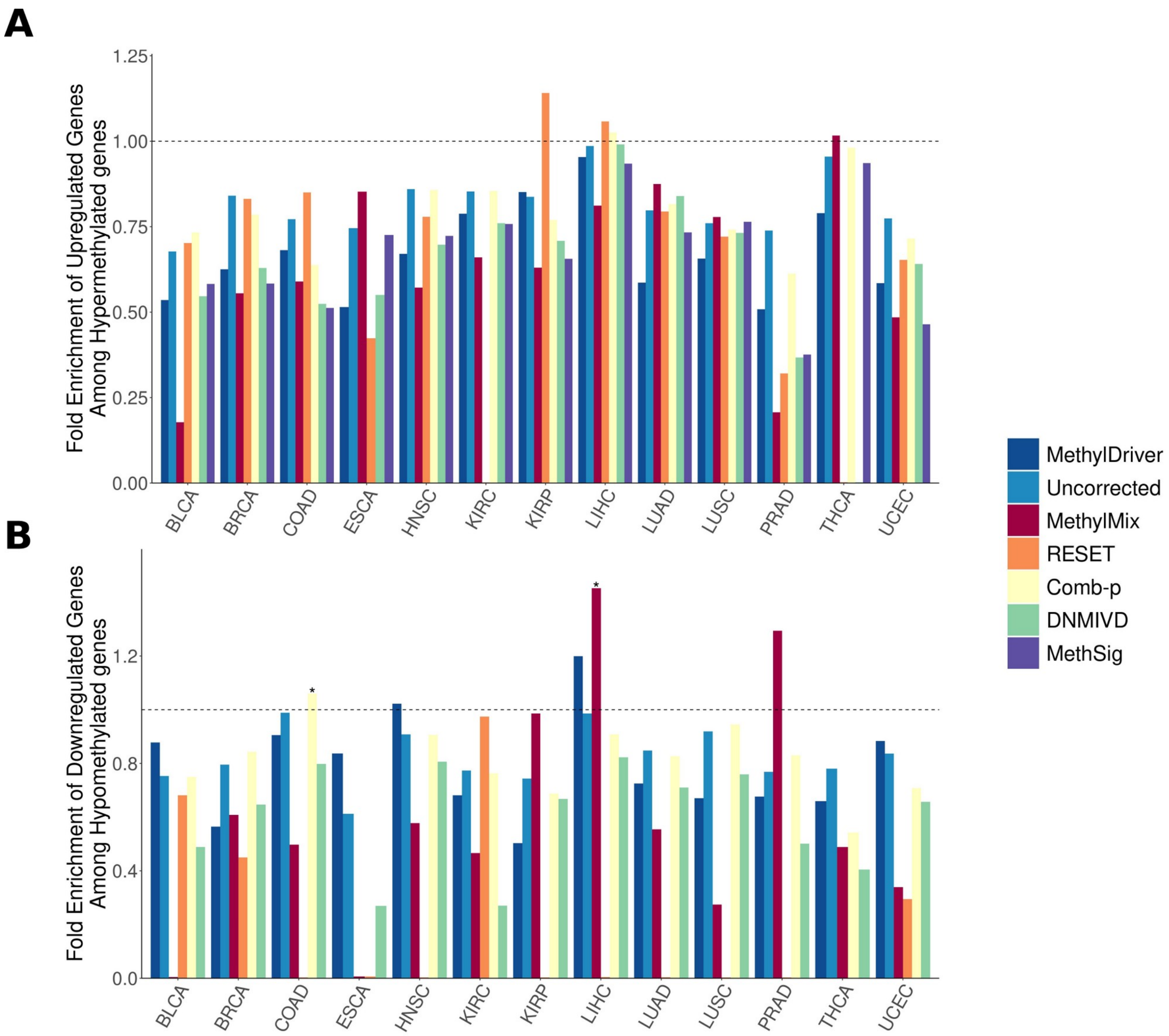

Supplementary Figure S3: Enrichment of upregulated genes among hypermethylated genes (A) and enrichment of downregulated genes among hypomethylated genes (B) for the different tools.

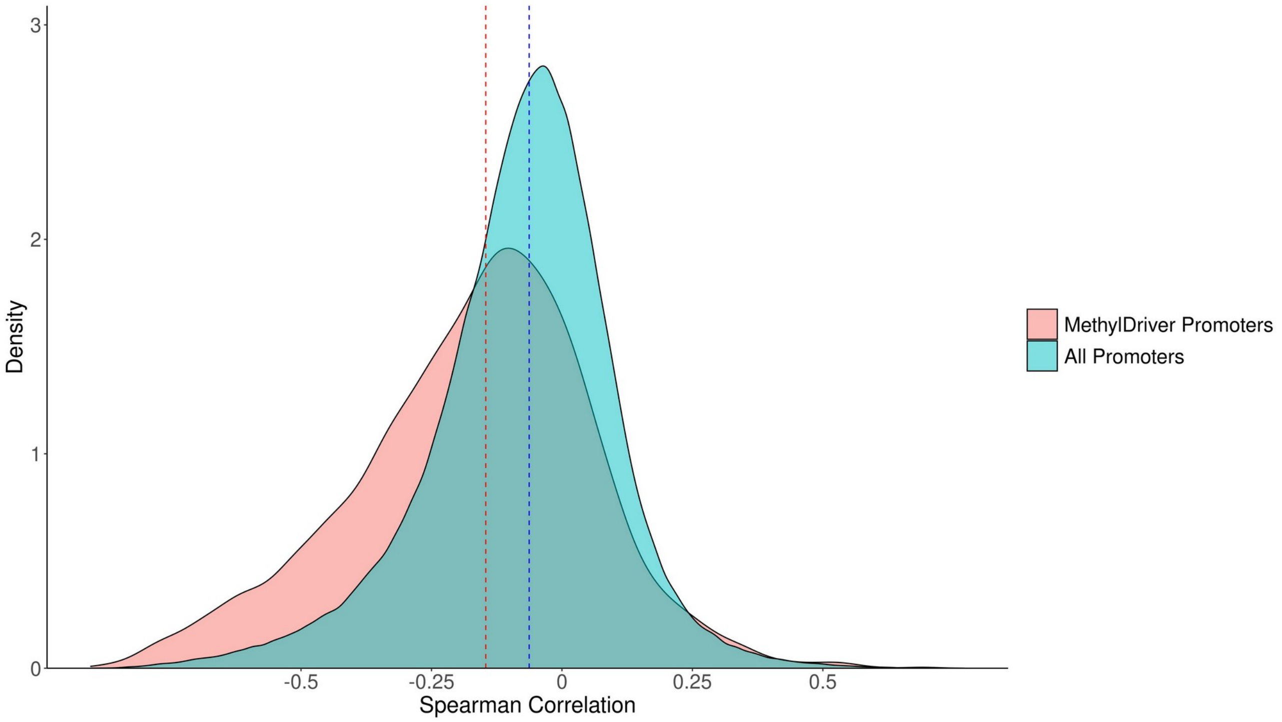

Supplementary Figure S4: Distribution of Spearman's rank correlation coefficient values between promoter methylation and TPM values for the corresponding gene for all promoters and those detected as being differentially methylated in cancer by MethylDriver. The blue and red dashed lines show the median correlation values for all promoters and the differentially methylated promoters, respectively.

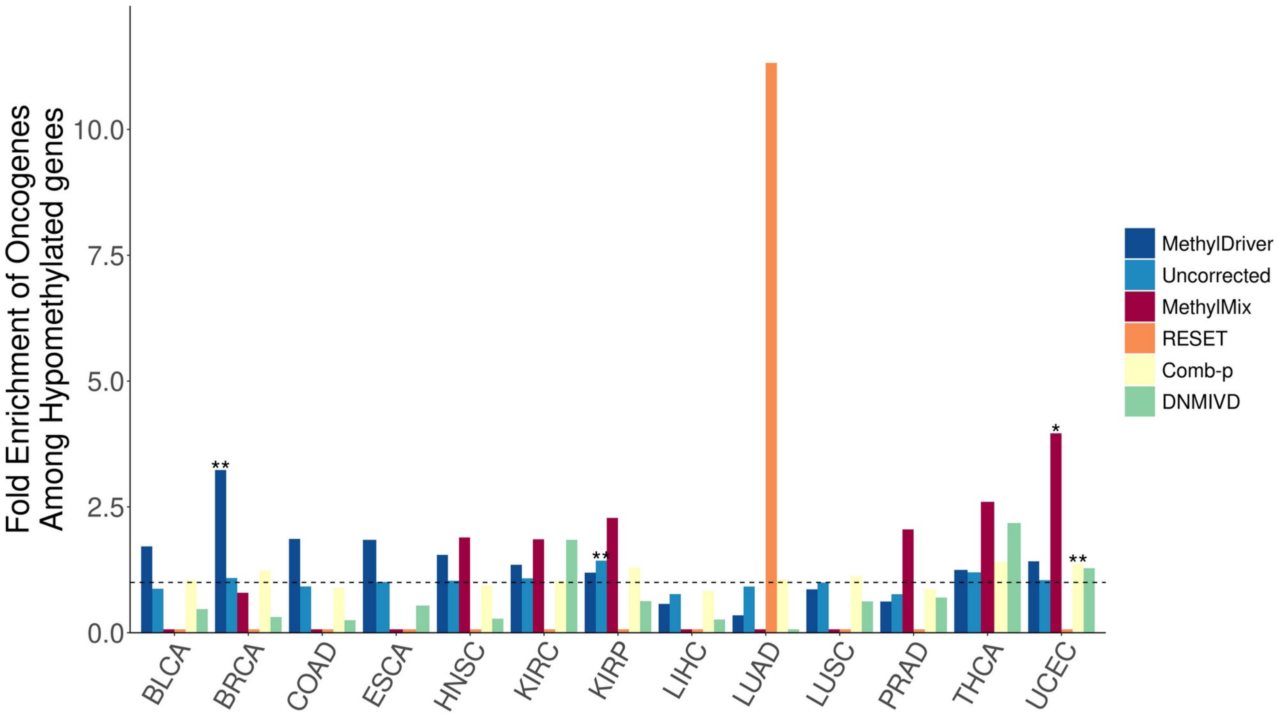

Supplementary Figure S5: Fold enrichment of oncogenes among hypomethylated genes detected by different tools.

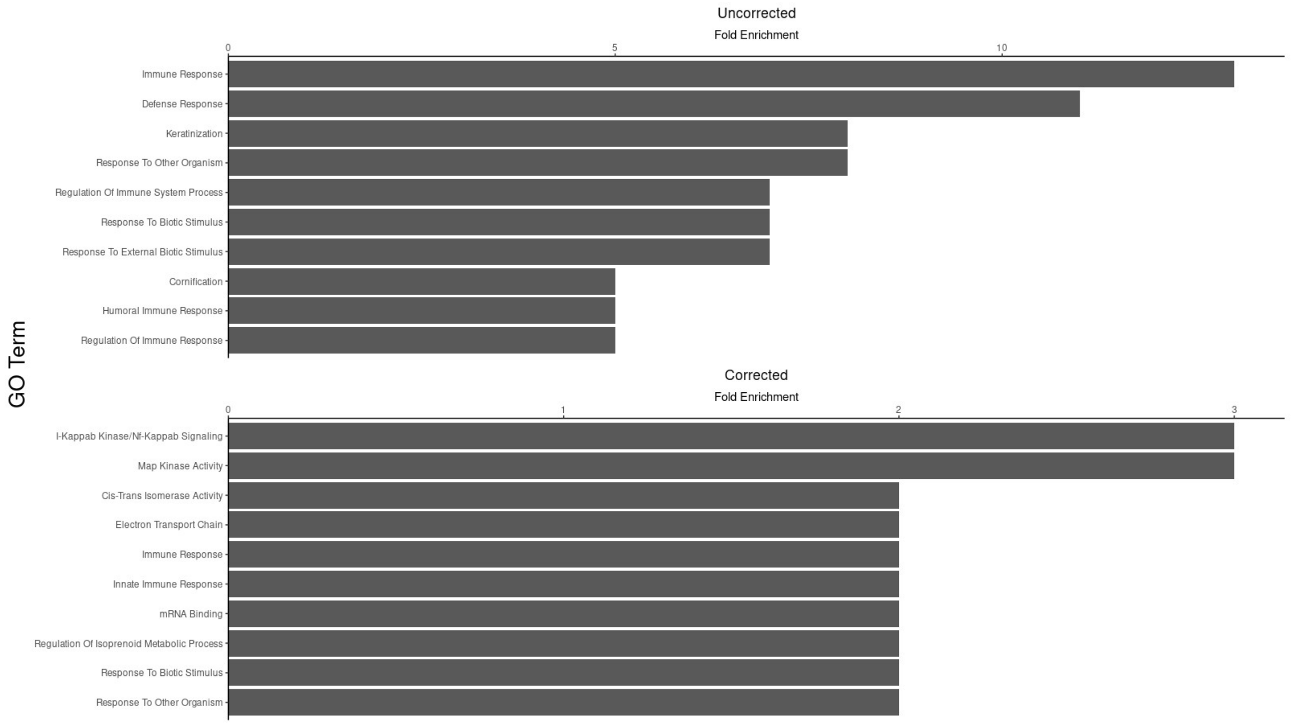

Supplementary Figure S6: GO term enrichment in corrected and uncorrected hypomethylated genes.

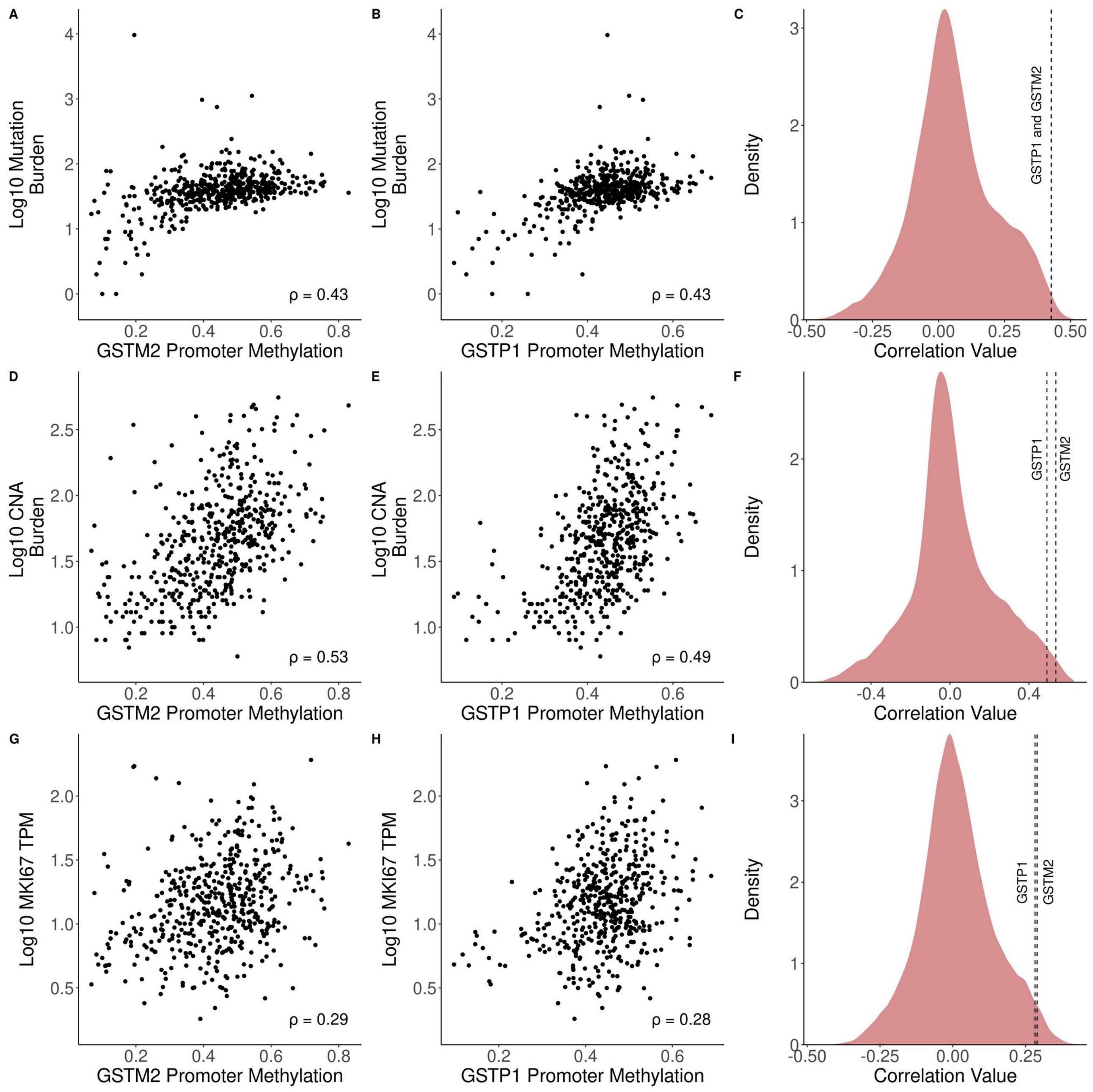

Supplementary Figure S7: Promoter methylation of GSTM2 and GSTP1 is associated with higher mutation burden and higher copy number alteration burden in prostate cancer. A and B) Correlation of GSTM2 and GSTP1 promoter methylation with the log of the tumour mutation burden. C) Location of GSTM2 and GSTP1 correlation values among the distribution of correlation values for methylation of all promoters with mutation count. D and E) Correlation of GSTM2 and GSTP1 promoter methylation with the log of the tumour copy number alteration burden. F) Location of GSTM2 and GSTP1 correlation values among the distribution of correlation values for methylation of all promoters with copy number alteration count. G and H) Correlation of GSTM2 and GSTP1 promoter methylation with the log of MKI67 TPM. I) Location of GSTM2 and GSTP1 correlation values among the distribution of correlation values for methylation of all promoters with MKI67 TPM. Correlation values are Spearman's rho.

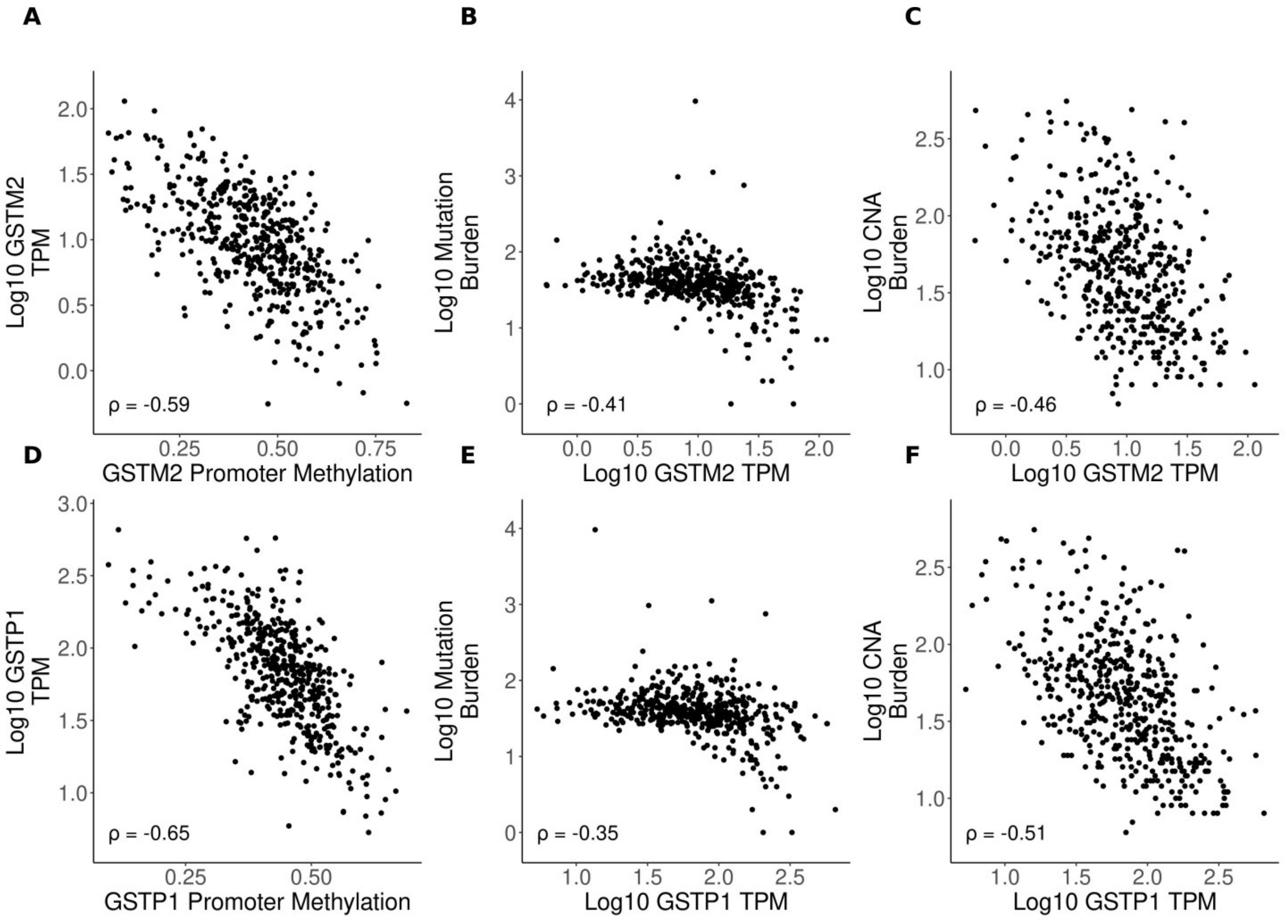

Supplementary Figure S8: Correlations of GSTM2 and GSTP1 promoter methylation with their expression levels (A and D) and of their expression with mutation burden (B and E) and CNA burden (C and F). Correlation values are Spearman correlations.
