# Supplementary material for: DNA methylation variation along the cancer epigenome and the identification of novel epigenetic driver events
Source: Nucleic Acids Res. 2021 Dec 6;49(22):12692–705. doi: 10.1093/nar/gkab1167 (PMC8682778; doi:10.1093/nar/gkab1167)
Supplement: gkab1167_Supplemental_Files [file gkab1167_supplemental_files.zip › supplementary_table_legends.docx]

Supplementary Table S1: Promoter genomic coordinates, associated genes and overlapping Infinium 450K probes

Supplementary Table S2: Unexpressed genes for each cancer type

Supplementary Table S3: Oncogenes and TSGs

Supplementary Table S4: Hypermethylated genes identified by MethylDriver in each cancer type

Supplementary Table S5: Hypomethylated genes identified by MethylDriver in each cancer type

Supplementary Table S6: Significant GO terms in PRAD
